# Structural Origins of Poor Health Outcomes in Documented Temporary Foreign Workers and Refugees in High-Income Countries: A Review

**DOI:** 10.3390/healthcare11091295

**Published:** 2023-05-01

**Authors:** Borum Yang, Clara Kelly, Isdore Chola Shamputa, Kimberley Barker, Duyen Thi Kim Nguyen

**Affiliations:** 1 Faculty of Medicine, Dalhousie University NB, Saint John, NB E2L 4L5, Canada; 2 Department of Nursing & Health Sciences, University of NB, Saint John, NB E2L 4L5, Canada; 3 Department of Public Health, Government of NB, Saint John, NB E1A E9H, Canada; 4 Faculty of Business, University of NB, Saint John, NB E2L 4L5, Canada

**Keywords:** structural discrimination, structural factors, health disparities, healthcare access, refugees, documented temporary foreign workers

## Abstract

Despite growing evidence of racial and institutional discrimination on minoritized communities and its negative effect on health, there are still gaps in the current literature identifying health disparities among minoritized communities. This review aims to identify health barriers faced by relatively less studied migrant subgroups including documented temporary foreign workers and refugees residing in high-income Organisation for Economic Co-operation and Development (OECD) countries focusing on the structural origins of differential health outcomes. We searched Medline, CINAHL, and Embase databases for papers describing health barriers for these groups published in English between 1 January 2011 and 30 July 2021. Two independent reviewers conducted a title, abstract, and full text screening with any discrepancies resolved by consensus or a third reviewer. Extracted data were analyzed using an inductive thematic analysis. Of the 381 articles that underwent full-text review, 27 articles were included in this review. We identified housing conditions, immigration policies, structural discrimination, and exploitative labour practices as the four major emerging themes that impacted the health and the access to healthcare services of our study populations. Our findings highlight the multidimensional nature of health inequities among migrant populations and a need to examine how the broader context of these factors influence their daily experiences.

## 1. Introduction

The proportion of international migrants has risen over the past five decades worldwide, with an estimated 272 million people living in a country other than their country of birth [1]. Notably, countries such as Canada have adopted immigration as a key means to support population, economic, and cultural growth [1,2]. For instance, Canada issued over 98,310 documented temporary foreign worker permits in 2019, and resettled 30,087 refugees, the highest number of any country worldwide [2].

Evidence of the differential health outcomes of migrants compared to the native-born population have long been well-established in both the United States and Canada [3,4].

In fact, the phenomena of racial and institutional discrimination on minoritized communities and its negative effects on health has been documented as early as the 1990s when the structural origins of poor health outcomes (e.g., poor regulation protections from pesticides) were identified [5]. However, despite growing evidence, public discourse and the medical literature community has been slow to identify structural racism as a root cause of health disparities [6,7]. This is certainly true regarding many reviews and research published to date. For example, Parajuli and Horey [8], Lane et al. [9], and Cheng et al. [10] revealed that more often than not, major themes regarding barriers to healthcare access pointed to individual characteristics and cultural factors (e.g., nutrition deficits, families’ conceptualization of life in Canada, and health literacy) as the primary drivers of disparities. Even the identified systemic barriers are limited to factors such as a lack of care-provider cultural competency, service time constraints, and policy issues regarding funding limits and the inadequate design of the healthcare system. Moreover, there are acknowledged gaps for subgroups of migrants; specifically, Chowdhury et al. [11] and Salami et al. [12] reported a dearth of literature on the health of migrants with disabilities and of documented temporary workers outside the agricultural sector.

The overarching goal of this study was to identify health barriers faced by relatively less-studied migrant subgroups including documented temporary foreign workers and refugees with a particular focus on the structural origins of differential health outcomes. These data will be used to inform the development a proposed newcomer health clinic in Southern New Brunswick, Canada, aimed to provide improved healthcare access to newcomers. Due to the limited literature available within Canada, we broadened the scope to documented migrants residing in high-income Organisation for Economic Co-operation and Development (OECD) countries. We also focused on research published in the past decade to provide an update on the literature. For the purposes of this review, the term structural factors is used to describe the term structural racism as conceptualized by Krieger [13] (p. 645): “the totality of ways in which societies foster discrimination, via mutually reinforcing [inequitable] systems… (e.g., in housing, education, employment, earnings, benefits, credit, media, healthcare, criminal justice, etc.) that in turn reinforce discriminatory beliefs, values, and distribution of resources”.

## 2. Materials and Methods

### 2.1. Study Design

Thus, to provide timely access to evidence-based health information, we used a review methodology guided by the Cochrane Handbook for systematic Reviews of Interventions [14]. This included defining a research question, conducting a search based on predetermined eligibility criteria and search strategy, selecting articles, extracting data, and a knowledge synthesis. A protocol was not registered for this review. A preliminary review of the literature was conducted to identify a list of key terms used to describe our sample and phenomenon of interest. (See Supplemental Materials (Figure S1) for the full list of key terms in the search string). Using the key terms identified, we conducted a comprehensive search to help inform the scope of pre-existing literature and find gaps in knowledge. The PICoS framework (Table 1), an alternative version of PICO modified for qualitative studies, was used to design the research question and the inclusion and exclusion criteria [15].

### 2.2. Search Strategy

The search strategy included a combination of subject headings and synonyms for each category under the PICoS framework (see Supplemental Materials for the full search strategy). The criterion for selecting the search terms was to include the preselected articles found in our preliminary search. An extensive list of terms was used to ensure the search was comprehensive. We searched three electronic databases, Medline, Cumulative Index of Nursing & Allied Health Literature (CINAHL), and Embase, based on their ready access to evidence-based content on the research topic, and robust search capability with controlled vocabulary and keywords. Searches were conducted on 19 July 2021 with a date limitation of 1 January 2011 to July 2021.

### 2.3. Inclusion and Exclusion Criteria

Qualitative and quantitative peer-reviewed primary studies published from 1 January 2011 to July 2021 were chosen to give priority to the more recent knowledge synthesis and to ensure a wide range of papers could be included, due to the lack of research in this area. Other inclusion criteria included research with (1) peer-reviewed articles written in English, (2) conducted in a high-income OECD country, and (3) whose study population of interest was documented temporary foreign workers and/or refugees. We chose to include studies looking at both the perspectives of the population in question or actors working in the field of migrant health (see Appendix A for actors working in the field of migrant health). We also chose to only include papers whose topic of interest was primarily health barriers and excluded those that did not have a specific focus on health barriers. We focused on articles written in English due to the language capabilities of the authors. In addition, we only included articles from high-income OECD countries to allow a better comparison to the country in which the results of this paper will be used (Canada). Papers that were not primary research, such as reviews, presentations, and editorial articles, were excluded to ensure rigor. Additionally, papers were excluded if the sample population was listed as a general migrant population or did not specify if it included other groups such as economic immigrants, nondocumented migrants, or groups not included in our population of interest. The full list of inclusion and exclusion criteria is presented in Table 2.

### 2.4. Data Selection

**Figure 1:**
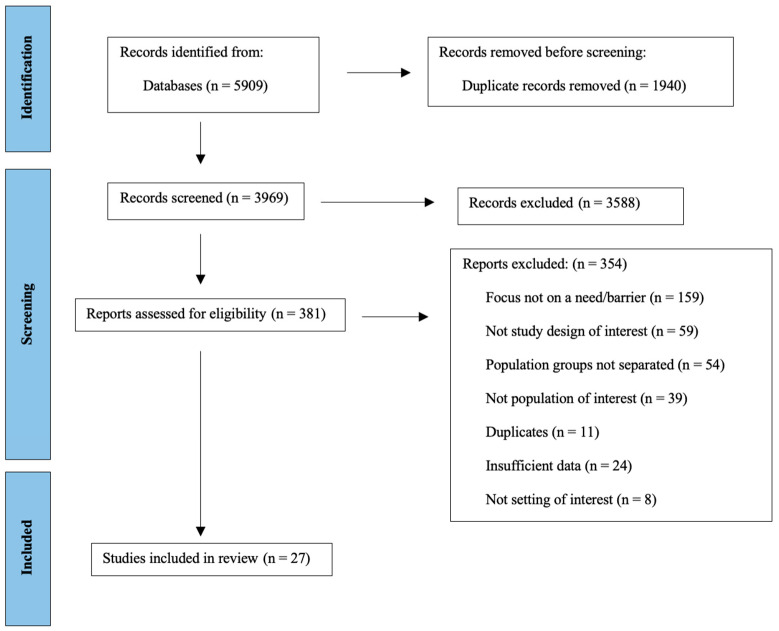
PRISMA flowchart of paper inclusion.

A search of the electronic databases identified a total of 5905 articles. Figure 1 outlines the selection process of articles as per the PRISMA guidelines. All search results were imported into Covidence. After removing duplicates (n = 1940), 3969 articles were identified. A pilot exercise using the same 10 abstracts and full-text articles was conducted to calibrate and ensure a standardization between author 1 and author 2. Then, author 1 and author 2 independently performed both title and abstract screening and full-text review; 277 conflicts were identified, which were resolved either by discussion or a third reviewer; the inter-rater reliability rate was 90.4% (3588/3969). Of the 381 articles that underwent full-text review, 27 articles were included in this review.

### 2.5. Data Extraction and Analysis

We developed an extraction tool adopted from the Cochrane handbook [14]. Extracted data included authors, publication year, study design, study location, research question, participant characteristics, size, and main findings (Appendix A). To systematically identify and synthesize findings across all included studies, we used a thematic analysis [16]. We used NVivo 12 Pro software to carry out the coding process. The first phase of analysis included familiarization, during which each paper was read multiple times, and any potential data of interest regarding health barriers were highlighted. For qualitative studies, the data of interest included interview quotations and paraphrases regarding barriers or needs our study population experienced in accessing healthcare. We included the perspectives of actors working in the field of migrant health to provide additional perspectives. Actors working in the field of migrant health included physicians, nurses, allied healthcare professionals, policymakers, advocacy agency workers, and others. The full list of the actors working in the field of migrant health can be found in Appendix A. For quantitative studies, the data of interest included variables significantly associated with the health status of the sample populations. Once this was done for all papers, the identified extracts were analyzed and labeled with a “code” that provided a definition or an interpretation. This process was repeated until all the papers were fully coded. The second phase comprised exploring different ways to combine the codes by drawing thematic maps and searching for unifying themes and patterns across the entire data set. Priority was given to themes addressing the structural origins of poor health outcomes. This was done for each population separately and additionally across both populations to identify the existence of similarities or differences among the two groups.

## 3. Results

### 3.1. Study Characteristics

Of the 27 papers included, 18 focused on refugee challenges, and 9 on documented temporary foreign worker challenges (see Table 3). Most studies were published from 2017 onwards (n = 22). Studies were mostly descriptive in nature, using a qualitative (n = 21), quantitative (n = 1), and mixed-methods (n = 5) approach. Most studies were conducted in Canada (n = 13), followed by the United States (n = 6), Australia (n = 3), New Zealand (n = 2), and one each from the Netherlands and Germany. Most studies discussed multiple ethnicities and presented them from various perspectives. Below, we describe in further detail the main barriers each group faced. Excerpts from selected papers demonstrating the emerging themes reported herein are presented in Table 4.

### 3.2. Barriers for Documented Temporary Foreign Workers

We identified nine studies in total that looked at documented temporary foreign workers; four studies examined caregivers, two examined agricultural workers, and one on various industries including meat processing, food services, hospitality, and construction. Two studies interviewed the perspectives of key informers. We discuss the four identified barriers below: housing conditions, immigration policies, structural discrimination, and exploitative labour practices.

#### 3.2.1. Housing Conditions

Our results showed that documented temporary foreign workers across different fields lived in suboptimal and unsafe conditions, including overcrowded housing, basements, and rooms without beds or windows [17,19,20,26]. Despite the removal of the “live-in” requirement in 2014 by the Canadian government, most caregivers still lived with their employers either because of the preference of the employers or the workers could not afford the high cost of living alone [27]. Caregivers noted significant distress related to the lack of privacy from an inability to lock doors to their bedrooms, being monitored by security cameras, and being hassled by the children of their host families [17,28] There was significant psychological stress noted because of the lack of socialization due to long work hours, remoteness of the homes, and limited transportation [18,26].

#### 3.2.2. Immigration Policies

A very common barrier identified for documented temporary foreign workers was regulations and government policies that placed them in precarious positions that directly impaired their access to care. Advocates and healthcare workers reported documented temporary foreign workers hiding their illness or choosing not to seek medical care to maintain a clean medical record necessary for a successful immigration application [17,29]. We found that many workers were aware of the exploitative working conditions but chose to accept them as they viewed obtaining permanent residency as a way towards a better future for their families [18].

The vulnerability of migrant workers to exploitative conditions was largely enabled by the absence of sufficient oversight and enforcement by regulatory bodies. Seasonal agricultural workers reported that workplace inspections were announced to the employers beforehand, which allowed hazardous and poor working conditions to continue unchecked [18,20]. Caregivers expressed that the lack of regulations exacerbated the uneven power dynamic between employer and caregiver, leaving them no choice but to tolerate hazardous work [18]. A number of immigrant service providers as well as policymakers noted that while policymakers expect the employers to take care of health needs of their workers, there is no policy mandating them to provide such services [17]. This was apparent in the numbers: despite a federal government requirement that employers are responsible for providing third-party health insurance for employees during their first three months, only 24% of caregivers in a study were found to have one [27].

In our review, only one group of documented temporary foreign workers reported significantly different experiences. Workers in the meat processing industry reported that the union provided them with an opportunity to advocate for their needs and have them resolved [20]. The union representative stressed the need for unions among documented temporary foreign workers because their precarious position left them vulnerable to exploitation [20].

#### 3.2.3. Structural Discrimination

Our final theme regarding barriers for documented temporary foreign workers includes structural discrimination. Workers reported having limited access to social and public spheres of life due to various forms of cultural and systemic exclusions. They reported experiencing intolerance from employers and struggled with expressed feelings of “cold indifference” despite efforts to be recognized as members of the community [28] (p. 212). Workers reported experiencing racial profiling during contact with law enforcement [23]. Caregivers reported experiencing isolation and alienation due to deskilling and downward social mobility experienced in their host countries, evidenced by facing verbal abuse from employers who harbored negative stereotypes [17,28,29].

#### 3.2.4. Exploitative Labour Practices

The most common barrier for documented temporary foreign workers examined was exploitative labour practices. We found that working conditions across all groups of workers were hazardous and unsafe, often involving contract breaches related to working hours and job descriptions. Workers were often asked to work through breaks, work without proper equipment, work overtime hours without pay, and perform tasks beyond their responsibilities and skills [17,19,20,26,27,28]. Notably, seasonal agricultural workers and caregivers reported long work hours as a major barrier to accessing health services when needed [17,20,26]. Employers were often found to use threats of job termination and deportation as leverage to maintain exploitative labor conditions [17,18,19,20,29,30]. Agricultural workers and healthcare workers reported being persuaded against or actively prevented from seeking medical care or accessing worker’s compensation by employers worried about incurring costs, with some even being harassed for reporting an injury [18,19,20,30].

### 3.3. Barriers for Refugees

We identified 18 studies regarding refugees: 5 studies looked at the perspectives of refugees; 8 studies considered the perspectives of actors working in the field of migrant health including but not limited to physicians, nurses, allied healthcare professionals, policymakers, and advocacy agency workers; and 5 studies examined both the perspectives of refugees and the actors working in the field of migrant health. The three main themes were: housing conditions, immigration policies, and structural discrimination.

#### 3.3.1. Housing Conditions

Another common health barrier we found was poor housing conditions. Results showed that the social context of migrants negatively impacted the refugees’ health by limiting access to social and economic opportunities. Refugees expressed a fear of hearing and witnessing gunshots, drug activities in the neighbourhood, and animals in their houses, which not only distracted them from tending to their health but also prevented them from venturing outside their homes [21]. In instances where refugees were located far from services, many reported ignoring their illness because they were unable to navigate the city transportation [25,31,32] Information from the literature indicated that refugees with disabilities were rarely provided with adequate accommodations related to their needs such as housing with elevators for those in [33]. This in turn limited their ability to access services such as in-person language classes and healthcare facilities offered outside of their homes [33].

#### 3.3.2. Immigration Policies

A common barrier identified by both refugees and actors working in the field of migrant health was immigration policies, specifically policies that discouraged or delayed care from taking place. Refugees and resettlement agencies described the process of applying for provincial health insurance as lengthy and difficult, which resulted in long periods of refugees being uninsured and unable to receive medical care [22,34]. They also reported having trouble finding clinics and providers who accept refugee medical assistance and reported the complexity of insurance as a barrier present at each point of access in the healthcare system [35]. Healthcare providers expressed significant challenges navigating the excessively complicated and constantly evolving policies that were hard to understand and keep track of [23,24,36]. The lack of institutional support and timely dissemination of information put the onus on individual providers to learn the workings of the complex system to ensure that their patients received appropriate medical care [36,37,38].

Healthcare providers described the healthcare system as being “reactionary” and ill-prepared to provide adequate care for the refugee population [38] (p. 5). Defragmented services resulted in poor information flow, which impeded the providers’ ability to ensure timely care and a continuity of care [37,39,40]. The underuse of available interpreter services was another significant barrier expressed by refugees, providers, and resettlement agencies [31]. Without the means of communication, refugees were sent home without being seen or left the pharmacy without understanding their prescriptions [32,36,41]. Family or community members more proficient in the language were seen to aid during instances without interpreters; however, this tended to compromise the quality of care due to faulty translations or issues disclosing private health information [25,38,42].

#### 3.3.3. Structural Discrimination

Finally, our third most common health barrier identified by refugees was structural discrimination. Our findings show that prejudice and discrimination by the public and among care providers serves as a major barrier. Healthcare workers expressed that the negative images of refugees in the media instigated a general disposition of the public to be rude towards refugees, and that the negative attitudes also extended to the care providers [37]. Refugees reported instances of not being able to make appointments because the receptionist would hang up on the phone from not understanding them [31]. They also reported not feeling respected or valued by the healthcare system, as observed during instances where refugees experienced significant stress related to not being provided with interpreters or explanations about decisions being made about their care [25]. There were reports of healthcare workers becoming angry or making blatantly racist remarks toward refugees [25]. Discrimination was often seen in the form of a dismissal of patients because of limited communication, considering their concerns as trivial, or a dismissal of mental health concerns as resettlement stress [34,43]. In fact, refugee resettlement workers described the whole mental health system as being unavailable to non-English speaking individuals [34].

## 4. Discussion

The purpose of this review was to bridge the gap in the current literature by identifying health barriers faced by documented temporary foreign workers and refugees in high-income OECD countries through a focused lens on the structural origins of poor health outcomes, and to gather information to help guide the planning of a proposed newcomer health clinic in Southern New Brunswick, Canada. We discuss four common emerging themes identified for both documented temporary foreign workers and refugees, while highlighting results that confirm previous findings as well as novel ones deserving attention. Further, we discuss the implications of these findings on their use in healthcare practice and plausible future directions of research.

### 4.1. Housing Conditions

The link between suboptimal housing and poor health outcomes, including stress, depression, and asthma exacerbations is well established in the scientific literature [44]. Our results showed that documented temporary foreign workers and refugees experienced poor housing conditions related to overcrowding and poor hygiene, similar to prior reports [12]. Specifically, new findings from our review demonstrated that previously unstudied populations including documented temporary foreign workers in nonagricultural sectors, as well as refugees with disabilities, also identified poor housing as a key determinant of health. Moreover, this review highlighted notable differences within migrant groups that are important to distinguish in order to better develop interventions including a lack of privacy from employers among caregivers versus a lack of bedding and hygiene among agricultural workers.

### 4.2. Immigration Policies

A growing literature points to the impact immigration policies have on health outcomes [4,6]. Historically, created to define a national belonging and ethnicity, many immigration policies have continued to restrict the rights of migrants [4]. These include discouraging individuals from participating in paid work and denying them protection against unemployment or injuries [45]. Findings from our review demonstrated the direct effects of policy on the health of migrant communities.

In concert with previous studies, immigration policies that required a clean medical record were seen as preventing those desiring permanent residency from seeking care. Our review further explored the detailed implications of such policies, including significant delays in the process of applying for health insurance resulting in long lapses in time where refugees were ineligible for medical care. These results have significant implications and calls for a closer look at the absence of appropriate oversight by institutions that result in migrant workers being vulnerable to abuse and exploitative work conditions. Healthcare workers can also be empowered to advocate for this population in knowing this information and can further advocate for better healthcare access and may inform future policy change considerations.

Our review found a “disconnect” between service agencies and the absence of well-established referral pathways as a major deterrent for the care provision for refugees. In addition, the lack of timely dissemination of information regarding policy changes to care providers was seen as a major constraint in providing timely care. These findings go beyond previous reviews that identified barriers limited to factors within the healthcare system. Our review highlighted how immigration policies outside the healthcare system, society, and institutions in general had a direct impact on creating barriers. This finding corroborates the findings from Bailey et al. [6] (p. 1458) that states the danger of “view[ing] these problems solely as a matter of institutional and interpersonal discrimination within health-care settings” as well as the need to “understand the broad context within which health-care systems operate” (p. 1458) such as intersectoral work guided by transdisciplinary frameworks. Policymakers, shareholders, advocates, and healthcare providers can benefit from knowing this disparity and work to improve intersectoral communication. Our findings outline how creating better pathways to access care can improve health outcomes.

A noteworthy and beneficial finding of this review includes the underuse of available language interpretation services by healthcare providers. Further investigations on the nature of the low uptake in routine clinical care would benefit hospitals and policymakers in ensuring an effective implementation of this important service. In addition, the knowledge of the lack of language services available to patients is important for healthcare providers to be aware of in order to better advocate for services.

### 4.3. Structural Discrimination

Scholars describe a process of “othering” in which minorities are ascribed a lower status on the social hierarchy due to perceived racial and ethnic differentials [4] (p. 2101). People lower on the social hierarchy not only have limited access to social goods but also experience a physiological stress response that increases the risk of chronic diseases [45]. Several papers in our review highlighted the way migrant workers and refugees are regarded and treated as subcitizens in their host countries. This was evident through the marginal living of migrants and a general lack of regard for their health and safety echoed in many of the studies. Acknowledging the role of cultural exclusion and racism would be critical in taking the next step to improving migrant health, as a growing body of literature point to a significant association between racism and poor mental and physical health [46]. Future research may elaborate on the types of microaggression within the healthcare system and how it affects the quality of care migrants receive.

### 4.4. Exploitative Labour Practices

We identified a broader variety of worker occupations, including caregiver, food industry, construction, and hospitality workers. Evidence suggests that job insecurity, occupational hazards, and having no control over high workloads lead to a range of physical and mental afflictions [45]. These findings have implications for the population’s health, as these factors influence health outcomes. Awareness of the challenges faced by temporary foreign workers would allow healthcare providers to serve the population better and have a greater understanding of their needs. In our review, many workers across the board endured hazardous and unsafe working conditions. Large meat-processing factory workers were the only unionized group who reported better experiences because of the advocacy provided by the union. Our findings revealed that little is known about the presence of unions in other parts or industries in Canada. It would be interesting to conduct further research into how and why certain industries were able to establish a union, as well as further characterize how it benefits the health outcomes of documented temporary foreign workers. The present findings also indicate that the current knowledge on the health of documented temporary foreign workers is limited to those surrounding their labor practices and work experiences, with a dearth of studies exploring their use of healthcare services or their experiences in the healthcare settings. Whether that is due to a lack of use or research is an issue for future work to explore.

### 4.5. Limitations

Our review includes several limitations. First, although our study provided a comprehensive review of the experiences of documented temporary foreign workers and refugees in the last decade, our findings should be interpreted with the awareness that it combined studies from multiple high-income OECD countries, with variable policies, societal norms, and healthcare systems. Second, the study focused on peer-reviewed articles written in English, which might have excluded data from studies written in non-English languages. Third, the inclusion of both qualitative and quantitative research designs, although done to increase the scope of reviewed literature, may limit the interpretation of the findings. Fourth, due to the vast nature of the challenges experienced by immigrants, future research should consider conducting a systematic or meta-analysis review, which may provide further nuances and contextualize these findings. Additional future research topics may also include identifying proposed solutions to such barriers and expectant outcomes.

## 5. Conclusions

This review aimed to identify the health barriers faced by documented temporary foreign workers and refugees residing in OECD countries, with a particular focus on the structural origins of differential health outcomes. This was done as the existing literature highlighted the lack of recognition by the public and the majority of the research community in identifying structural origins as a cause of health disparities. In addition to corroborating prior reports, the present review identified structural racism as a direct contributor to poor health outcomes in these populations, as evidenced through studies conducted in the last decade. As there were acknowledged gaps in the literature for subgroups of migrants, we focused on those groups, namely, documented temporary foreign works and refugees. This review will contribute to the growing literature indicating the need to address systemic barriers faced by documented temporary foreign workers and refugees when accessing healthcare services. These findings may benefit healthcare providers, policymakers, and relevant stakeholders interested in developing and improving efforts to eliminate health inequity. In addition, the findings from this review will assist the authors in advocating to relevant authorities about reducing systemic barriers, as they create the proposed newcomer health clinic. Our findings can also inform the newcomer health clinic policies and procedures to make healthcare more accessible to temporary foreign workers and refugees.

## Figures and Tables

**Table 1:** PICoS Framework Identifying the Main Focus of Study.

Population	Phenomenon of Interest	Context	Study Type
Documented temporary foreign workers, refugees	Health barriers	OECD high-income countries	Peer-reviewed, primary research

**Table 2:** Inclusion and Exclusion Criteria for Selection of Papers Included.

Inclusion Criteria	Exclusion Criteria
OECD high-income country	OECD middle- or low-income country/non-OECD country
Documented temporary foreign worker, or refugee population	General or nonspecific migrant population (i.e., undocumented immigrants, asylum seekers, illegal migrants)
Focus of paper is health barriers, challenges, or needs	Paper focused on policy review, changes, or adaptations
Perspective of key informant or population in question	Papers focused on describing disease prevalence
Population of interest is the main focus of the paper	Papers focused on a topic other than healthcare needs, access, barriers, or challenges
Published in English	Papers published in a language other than English
Published between January 2011 and July 2021	Papers published before 2011
Primary research	Reviews, editorials, commentaries, letters, perspectives, news articles, presentations, conference abstracts, symposium summaries, or another type of nonprimary research

**Table 3:** Selected Excerpts Demonstrating Themes Identified in the Literature.

Documented Temporary Foreign Worker Excerpts	
Theme	Example
Housing conditions	“My house, the family that I live with, it’s stuffed with cameras…it is not like you do something bad to the kids but you have to all the time think about what you are doing—maybe they are not watching me but I think maybe they were; like my self-esteem goes very down and you feel, okay, no I’m not gonna do this thing because may be someone’s watching me” [17] (p. 5). Jose pays 800 dollars to live here. He suggests to the interviewer that a report be written, “a report of what the living conditions are here”. “The boss should out of obligation put a bed, a little one, for each worker. He doesn’t have [provide] anything. So I’m expected to throw myself on the ground” [18] (p. 214).
Immigration policies	The last thing they have in mind is their health issue. And when a certain health issue happen, they try to cover it, right? Because part of having a successful immigration application is having a positive medical exam. There’s a couple that we worked with recently, whose husband contracted a liver issue, and um, and they hid it from, you know (the doctors)? But when they were forced to take the medical exam from the doctor provided by the immigration, they failed. And that’s the basis of their denial … So the whole family is now facing deportation [19] (p. 17).
Structural discrimination	In Beaumont [fictional name], when we were in Beaumont, we couldn’t do [nothing]. If a black guy comes in the community or in the city or in the town of Beaumont—haven’t done anything—the cops would be coming straight to us. It was only 4 black guys […] and everyone, oh those are the Jamaicans [18] (p. 212).
Exploitative labour practices	I got into an accident, because they are supposed to change you every forty-five minutes or every hour to prevent any injury in your body. They are supposed to rotate all the personnel. But they left me there for four hours. So I started having a lot of pain from my neck to my back, and then my shoulder. And I was there for four hours so nobody was changing me … So I deal with them and say, you know what, I have to take some days off and [the manager of the company] told me, no, you can’t because we need you [19,20] (p. 437). I make thirty-two kidney stones, they took me to the hospital and I get surgery”. When he explained the situation to his boss and asked, without success, to work fewer hours in order to rest, “[h]e took my papers, threw them to the floor and said to me ‘your health is not my business’ …” [20] (p. 437).
**Refugee Excerpts**	
**Theme**	**Examples**
Housing conditions	We did not feel safe and secure here at all. When we first arrived we had shots fired on our home. We had to keep the windows and doors locked constantly. We did not dare to go out. Even when we needed to go out to get food, the kids would be too afraid to leave the house [21] (p. 5).
Immigration policies	‘‘I don’t know what is the reason that my daughter doesn’t have Medicaid at the current time, and since two months she is without Medicaid, and she is a child, and why the Medicaid stopped. We filled application for Medicaid two times. First time they asked us to fill it again, and second time before one week we sent another application, and there is no replay or interview appointment or any thing from Medicaid office yet” [22] (p. 1531). “And of course this makes it very difficult in everyday life, so if a doctor first has to think about what the Government agencies will approve and what they will not approve, then, yes, it can be difficult for the medical side of the process” [23] (p. 3). There’s a couple of key disconnects in the [government] system [that need to be] bridged in order to actually do systems level planning for the intake of refugees […] two key disconnects […] are the fact that refugee intake is a federal issue, [while] provision of health and social services is a provincial issue and they’re just not linked [24] (p. 152).
Structural discrimination	The doctor was an old man and when he see our ID [identification card], he said, “oh, from Sudan. Oh, Sudanese, they are the worst people” … I felt really bad. I’m a human like them. I have everything; the only thing that I cannot understand is language [25] (p. 5).

**Table 4:** Structural Barriers Identified in the Literature for Each Studied Population.

Challenge	Refugees	Temporary Foreign Workers
Housing conditions	X	X
Immigration policies	X	X
Structural discrimination	X	X
Exploitative labour practices		X

## Data Availability

The data that support the findings of this study are available from the first author (B.Y.), upon reasonable request.

## Supplementary Materials

The following supporting information can be downloaded at: https://www.mdpi.com/article/10.3390/healthcare11091295/s1, Figure S1: Search strings.

## Appendix A

**Table A1:** Data Extraction Table: Characteristics of Reviewed Papers.

Documented Temporary Foreign Worker Papers Included						
Author(s)	Year	Study Type	Study Location	Research Question	Sample Size	Main Findings
Bhuyan et al. [28]	2018	Mixed methods	Greater Toronto Area, Ontario and Calgary, Alberta, Canada	This study examined several questions including: how do migrant caregivers make sense of changes to the Canada’s caregiver policy in 2014? How do the chances impact their access to permanent residence? How does it shape their response to workplace abuse and exploitation?	33 caregivers under the Live-in Caregiver program, Caring for Children Program, or Caring for People with High Medical Needs Program. 31 from the Philippines	This study found that despite the potential benefit of removing the “live-in” requirement, conditions of the new policy reinforced broader immigrant trends that restricted access to permanent residence and produced longer periods of precarious status. The bureaucratic management of the program forced caregivers to go for long periods without work authorization, which contributed to the production of “illegality” due to increasing financial insecurity, vulnerability for abuse and exploitation of migrant caregivers.
Carlos and Wilson [27]	2018	Qualitative	Greater Toronto Area, Ontario, Canada	The paper sought to gain insight into perceived changes in health status and access to healthcare services related to employment among a group of Filipina live-in caregivers in the Greater Toronto Area.	21 documented temporary foreign workers (4 current live-in caregivers, 17 former live-in caregivers), all from Philippines.	The results of the study revealed that 43% of workers perceived a decline in health status postmigration, and 67% reported experiencing new physical and mental health issues. Four key aspects of caregiver work were seen to contribute to health: (1) Work responsibilities: caregivers reported that negative interactions with employers related to job duties and caring for children and elderly led to stress. (2) Long work hours: 52% of caregivers reported working more than 8 hrs on a regular basis. There were instances of contract breaches related to working hours. (3) Living in: caregivers reported an unhealthy work environment including a lack of privacy, freedom, and lack of control over diet. (4) Access to healthcare services: 52% of caregivers reported that work posed barriers to accessing health services. Despite this federal government requirement, only twenty-four percent (n = 5) of participants reported that their employers provided third-party health insurance. Remoteness of the workplace, lack of access to transportation, and long work hours were the most frequently identified barriers.
Caxaj and Cohen [26]	2019	Qualitative	British Columbia, Canada	This qualitative study intended to understand the workplace experiences of migrant agricultural workers as well as how those experiences influenced their health.	23 seasonal agricultural worker’s program workers, 1 key informant (volunteer support person) from Mexico, Jamaica.	The study revealed 2 over-arching themes that determined workers’ health: (1) Silencing by authorities: authorities intended to protect them, such as consular officials and employers, actively or passively helped reinforce coercive power dynamics and/or failed to deliver on protections promised under the Seasonal Agricultural Worker’s Program in Canada. (2) Precarious legal status: Workers felt compelled to sacrifice their physical health for short-term security in the SAWP, while at the same time endangering their long-term livelihood in this same program, and even their basic survival. Participants in this study were reluctant to report workplace injuries, refuse unsafe practices, seek medical attention for workplace injuries, and assert their rights to adequate hygiene.
Caxaj and Diaz [18]	2018	Qualitative	British Columbia, Canada	This paper intended to examine the experiences of temporary migrant agricultural workers pertaining to belonging and wellbeing in a rural setting within British Columbia, Canada.	17 documented temporary foreign workers (seasonal agricultural worker’s program workers) from Mexico, Jamaica.	The study revealed an overarching theme of marginal living among migrant workers. Workers reported having limited access to the social and public spheres of life due to various forms of cultural and institutional exclusion. These contributed to a sense of hopelessness, anxiety, depression, and suicide. They also struggled for their basic needs, frequently facing poor housing conditions, despite housing inspections, and access to necessary hygiene. With the limited social ties and lack of work/home division, workers were seen to be vulnerable to disrespectful behavior or negative interpersonal encounters.
Cedillo et al. [20]	2019	Mixed methods	Alberta and Manitoba, Canada	This study aimed to shed light on working conditions reported by documented temporary foreign workers in Canada, and to document hazards to which these workers were exposed and the challenges they faced in exercising their OHS (Occupational Health and Safety) rights.	22 documented temporary foreign workers (8 meat processing workers, 4 hotel workers, 6 food industry workers, 3 construction industry workers, 1 unknown)	The results revealed the presence of OHS hazards specific to sectors such as exposures to heavy workloads in the hospitality industry, breaching of work contracts in food services, and a lack of proper PPE and ergonomic hazards in construction. This study found that the workers’ migrant status and the employer’s role in applying for permanent residency was the main determinant for a worker’s inability to speak up against work and health violations. A key factor that showed a positive impact on protecting workers against abusive practices was the presence of an active union. Unionized workers in a meat-processing facility were seen to receive language support as well as assistance with filing taxes.
Cole et al. [30]	2019	Qualitative	Rural Southern Ontario, Canada	The purpose of this study was to explore rural health professionals’ perspectives on caring for migrant agricultural workers in rural Ontario, Canada.	108 actors working in the field of migrant health (rural primary care physicians, nurse, allied healthcare professionals)	The study identified structural and intercultural challenges in providing proper care for migrant agricultural workers: (1) Structural challenges: difficulty preventing work-related injuries; employers compromising confidentiality; worker’s fear of losing employment and return to countries of origin; lack of info about insurance coverage and workers’ living situations; scheduling conflicts between clinic hours and workers’ availability. (2) Intercultural challenges: language/communication barriers/ perspectives; limited professional knowledge of migrant agricultural workers.
Hill et al. [29]	2019	Qualitative	Fort McMurray, Alberta, Canada	This study looked to examine the occupational health and safety experiences of migrant workers employed as live-in caregivers in Fort McMurray, Alberta Canada.	120 documented temporary foreign workers (live-in caregivers)	The study revealed how various occupational hazards could be traced to the organization and conditions of work and how (im)mobility could intensify them. These included federal policies that tied legal status to employers and employment, blurred relations with employers that resulted in an unpredictable and elastic scope of work, and lack of appropriate oversight.
Salami et al. [19]	2018	Qualitative	Alberta, Canada	This study examined the perspectives of the actors working in the field of migrant health on the health and well-being of documented temporary foreign workers in Alberta, Canada.	13 actors working in the field of migrant health (7 social and immigrant service providers, 4 TFW advocates, and 2 policymakers)	The results of this study indicated that for documented temporary foreign workers, their immigration status was the single most important determinant of health. Their status determined which services workers had access to. Contributing factors were a lack of regulation, capacity, and willingness by employers to attend to workers’ health.
Vahabi and Wong [17]	2017	Mixed methods	Greater Toronto Area, Ontario, Canada	This study aimed to gain an understanding of the work-related experiences and mental health of migrant live-in caregivers in the Greater Toronto Area in Ontario, Canada.	30 documented temporary foreign workers (live-in caregivers) Philippines, Hungary, Ukraine, Poland	This study revealed a number of key themes relating to caregivers’ experiences: (1) Precarious migrant status that was linked to their employment status rendered workers powerless to respond to workplace abuse and exploitation. (2) The deskilling and downward social mobility reinforced the isolation and alienation of workers.
**Refugee Papers Included**						
**Author(s)**	**Year**	**Study Type**	**Study Location**	**Research Question**	**Sample Size**	**Main Findings**
Alwan et al. [21]	2020	Qualitative	Cincinnati, United States	This study sought to explore Syrian refugee parents’ beliefs, perspectives, and practices regarding their children’s health through in-depth interviews.	18 Syrian refugees	The study found that refugees reported experiencing stressors that precluded them from seeking care and maintaining their children’s health. Stressors were both environmental and psychosocial, including poor housing and neighborhoods as well as inadequate transportation and translation services.
Baauw et al. [39]	2018	Quantitative	Netherlands	The aim of the study was to gain insight into the barriers in the healthcare for refugee children perceived by pediatricians by analyzing logistical problems reported by pediatricians through the Dutch Pediatric Surveillance Unit.	1300 pediatricians	The study identified 74 cases of pediatrician-reported logistical problems during the care of refugee children. The barriers identified included policies and regulations that resulted in frequent relocations of refugee children. This led to missing health history, poor communication, and faulty medical handoffs that disrupted the continuity of medical care for refugee children.
Clark et al. [32]	2014	Qualitative	South Australia	This qualitative study sought to identify barriers to accessing primary healthcare services and explore medicine-related issues as experienced by refugee women in South Australia.	38 refugees	The results found that language was a key barrier in accessing healthcare for refugees. Refugees reported an inconsistent provision of interpreters, including many occasions when they did not ask for an interpreter and an interpreter was not provided for them. This often left the children or neighbors acting as informal translators.
Floyd and Sakellariou [25]	2017	Qualitative	Vancouver, British Columbia, Canada	This study explored the lived experiences of recently arrived, government-assisted refugee women, who were nonliterate and non-English-speaking when they arrived in the country, who attempted to access healthcare services.	8 refugees	The results illustrated the intersection of a limited knowledge of the local language with low literacy, gender, and refugee status and how it impacted women’s access to healthcare. Three themes identified from the results included dependence, isolation, and resourcefulness as factors influencing healthcare access.
Guruge et al. [43]	2018	Qualitative	Greater Toronto Area, Ontario, Canada	This study focused on exploring the healthcare needs of newcomer Syrian women, their experiences in accessing and using health services, and the factors and conditions that shaped whether and how they accessed and utilized health services in the Greater Toronto Area.	58 refugees	Results from the study included the identification of participant’s health concerns, which included chronic conditions as well as new and emerging issues. Factors enabling access to services included initial health insurance and coverage, whereas language and social disconnection were barriers. Additionally, it was identified that beliefs about naturopathic medicine, settlement in suburban areas with limited public transportation, and lack of linguistically, culturally, and gender-appropriate services negatively affected access to and use of healthcare services.
Hahn et al. [23]	2020	Qualitative	Germany	This qualitative study intended to explore the health experiences of refugees and the experiences of healthcare professionals and administrators involved in providing refugee healthcare.	12 refugees and 13 actors working in the field of migrant health (7 physicians, practice assistants and 6 “administrators involved in refugee matters”)	Legal, sociocultural, environmental, and communication aspects were the four main categories identified as crucial for equity in healthcare.
Kay et al. [41]	2016	Qualitative	Brisbane, Australia	This qualitative study focused on exploring the barriers to and facilitators of quality use of medicines as experienced by refugees, from the perspectives of pharmacists, general practitioners, and nurses working in the primary healthcare setting.	12 actors working in the field of migrant health (2 GPs, 3 RNs, 4 pharmacists, 3 refugee health leaders)	Barriers identified in the results included communication barriers, cultural barriers, limited health literacy, financial cost, and systematic barriers. Additionally, facilitators of access to medicines were identified and included better coordination between healthcare providers, community engagement, providing appropriate education, and improved healthcare provider training.
Mirza and Heinemann [33]	2011	Qualitative	Midwestern United States	This qualitative study sought to examine the adequacy of service systems that existed for addressing disabled refugees’ needs who had resettled in the United States.	15 refugees and 10 actors working in the field of migrant health (3 senior staff members at local resettlement agencies, 2 workers in refugee resource and advocacy agencies working on the international platform, 1 coordinator of refugee services at a local rehabilitation hospital, 1 staff at a local center for independent living, 3 staff at ethnic community-based organization)	Disabled refugee participants had unmet disability-related needs and poor access to resettlement resources due to their disabilities. There was a disconnect between disabled refugees and service providers, as the providers had minimal awareness about disability and lacked knowledge about the biomedical perspective, which led to mistrust, a lack of culturally safe care, and limited resources available to the refugees.
Newbold and McKeary [24]	2017	Qualitative	Hamilton, Ontario, Canada	This qualitative study explored the difficulties faced by local healthcare providers in providing care to refugee populations in the face of constantly evolving refugee policies, programs, and arrivals.	14 actors working in the field of migrant health (executive directors, program managers, nurses, physicians, health educators, settlement workers, and community health center employees)	Results identified new challenges faced by healthcare providers, including the difficulty of providing care and services to a diverse refugee population and the lack of knowledge associated with constantly evolving policies and programs.
Novak et al. [40]	2021	Mixed methods	Australia	This mixed-methods study aimed to describe the perceptions of healthcare workers working with refugee patients at a large metropolitan public health service with respect to how they identified, managed, and cared for their patients.	215 actors working in the field of migrant health (disciplines identified: 48 physicians, 85 nurses, 15 midwives, 50 allied heath clinicians)	The results identified potential service barriers which included insufficient identification, a lack of interpreter availability, education, healthcare worker capacity, and providing culturally specific treatment.
Ogunsiji et al. [37]	2018	Qualitative	Sydney, New South Wales, Australia	This qualitative study focused on exploring the experiences of the nursing workforce caring for refugees.	6 actors working in the field of migrant health (refugee health nurses)	Results identified three main themes including caring for clients with challenging needs, challenges in the course of caring for refugees, and passion in caring for refugees.
Pejic et al. [42]	2019	Qualitative	Colorado, United States	This qualitative study looked at understanding the training experience of psychiatry residents working with refugees and to assess the level of satisfaction of refugees who sought psychological treatment at the University of Colorado Hospital (UCH)’s Refugee Mental Health Program of Colorado (RMHPC).	10 refugees and 9 actors working in the field of migrant health (psychiatry residents)	Results consisted of five major themes that summarized the psychiatry resident’s experiences, including adapting practices to meet refugee needs, the value of supervision, cultural barriers, the need for extra resources, and the effect on future practice. Additionally, four major themes emerged summarizing the refugee’s experiences, including reasons for seeking treatment, barriers to treatment, a resident’s knowledge of culture and needs, and quality of treatment.
Richard et al. [38]	2019	Qualitative	Southern region of New Zealand	This qualitative study sought to explore the perspectives of primary healthcare professionals providing care to refugees through mainstream general practice.	15 actors working in the field of migrant health (9 general practitioners and 6 practice nurses)	The results illustrated three analytical constructs including a meaningful relational engagement with refugees, appropriate refugee healthcare delivery, and a provider’s professional development shaped by complexity.
Shannon et al. [34]	2016	Mixed methods	Midwestern states, United States	This mixed-methods study examined the characteristics of successful and unsuccessful mental health referrals with respect to what providers did across diverse practice settings.	64 actors working in the field of migrant health (social service provider, nonclinical social worker, case manager, care coordinator, mental health provider, nurse, primary care provider or nurse practitioner, school social worker)	Results separated the characteristics of both successful and unsuccessful referrals. Active care coordination, establishing trust, a proactive resolution of barriers, and culturally responsive care were facilitating successful referrals, whereas unsuccessful referrals were characterized by cultural barriers, a lack of care coordination, a refusal to see refugees, and system and language barriers.
Shrestha-Ranjit et al. [31]	2020	Qualitative	2 regions in New Zealand	This qualitative study explored the perspectives of Bhutanese refugee women who used interpreter services in primary healthcare settings after relocation to New Zealand.	40 refugees and 12 actors working in the field of migrant health (5 nurses, 4 general practitioners, and 3 midwives)	The results revealed inadequacies and constraints in the delivery of sociocultural and linguistically effective interpreting services to refugee women as well as provided evidence for recommendations to address these insufficiencies.
Vermette et al. [22]	2015	Qualitative	Dallas, Texas, United States	This qualitative study focused on identifying persistent barriers to healthcare access for Iraqi refugee children and potential solutions from the perspectives of parents and refugee service providers.	24 refugees	Results highlighted that provider availability, Medicaid maintenance and renewal, language issues, and inadequate recognition of post-traumatic stress disorder were barriers to care for the children of the Iraqi families.
Winn et al. [36]	2018	Qualitative	Calgary, Alberta, Canada	This qualitative study intended to understand the experiences of healthcare professionals caring for pregnant refugee women while taking into consideration recent contextual changes to the refugee landscape in Canada.	10 actors working in the field of migrant health (any healthcare professional who had experience caring for pregnant refugee women)	Several barriers when caring for pregnant refugees were described in the results, including language barriers, difficulty navigating the healthcare system, and cultural barriers such as managing traditional gender dynamics, only wanting a female provider, and differences in medical practices.
Zeidan et al. [35]	2019	Qualitative	Northeast United States	This qualitative study aimed to understand the barriers to accessing acute care by newly arrived refugees and identify potential improvements from refugees and resettlement agencies.	16 refugees and 12 actors working in the field of migrant health (employees from refugee resettlement/post-resettlement agencies)	Results reported several barriers to accessing acute care, including challenges in understanding the U.S. healthcare system, difficulty scheduling timely outpatient acute care visits, significant language barriers, and confusion over the complexity of health insurance.
