# Supplementary material for: Structural Origins of Poor Health Outcomes in Documented Temporary Foreign Workers and Refugees in High-Income Countries: A Review
Source: Healthcare (Basel). 2023 May 1;11(9):1295. doi: 10.3390/healthcare11091295 (PMC10177793; doi:10.3390/healthcare11091295)
Supplement: Supplementary file 1 [file healthcare-11-01295-s001.zip › healthcare-2294774-supplementary.pdf]

Supplemental Material A

Figure S1. Search strings

Medline Search String

1. Refugees/
2. refugee*.ab,kf,ti.
3. (asylum adj2 seeker*).ti,ab,kf.
4. "Transients and Migrants"/
5. (temporary adj2 foreign adj2 worker*).ab,kf,ti.
6. (foreign adj2 worker*).ab,kf,ti.
7. (migrant adj2 worker*).ab,kf,ti.
8. exp canada/ or united states/ or chile/ or israel/ or japan/ or Republic of Korea/ or austria/ or belgium/ or estonia/ or latvia/ or lithuania/ or czech republic/ or hungary/ or poland/ or slovakia/ or slovenia/ or france/ or germany/ or united kingdom/ or england/ or greece/ or ireland/ or italy/ or luxembourg/ or netherlands/ or portugal/ or denmark/ or finland/ or iceland/ or norway/ or sweden/ or spain/ or switzerland/ or australia/ or new zealand/
9. (canad* or "british columbia" or alberta or saskatchewan or manitoba or ontario or quebec or "new brunswick" or "nova scotia" or "prince edward island" or newfoundland labrador or nunavut or "northwest territories" or yukon).ti,ab,kf.
10. (high adj2 income adj2 countr*).ti,ab,kf.
11. Developed Countries/
12. (reception adj2 countr*).ti,ab,kf.
13. (resettlement adj2 countr*).ti,ab,kf.
14. Health/
15. (health* adj2 need*).ti,ab,kf.
16. (unmet adj2 need*).ti,ab,kf.
17. "Health Services Needs and Demand"/
18. "delivery of health care"/
19. culturally competent care/
20. health services accessibility/
21. Health Status/
22. health status disparities/
23. Healthcare Disparities/
24. "Needs Assessment"/
25. "Occupational Health"/
26. barrier*.ti,ab,kf.
27. challenge*.ti,ab,kf.
28. constraint*.ti,ab,kf.
29. OR/1-8
30. OR/9-13
31. OR/14-28
32. 29 AND 30 AND 31
33. limit 32 to (english language and yr="2011 -Current")

CINAHL Search String

1. (MH "Refugees")
2. TI refugee* or AB refugee*
3. TI asylum N2 seeker* or AB asylum N2 seeker*
4. (MH "Transients and Migrants")
5. TI temporary N2 foreign N2 worker* or AB temporary N2 foreign N2 worker*
6. TI foreign N2 worker* or AB foreign N2 worker*

7. TI migrant N2 worker* or AB migrant N2 worker*
8. (MH "Canada+") OR (MH "United States") OR (MH "Chile") OR (MH "Israel") OR (MH "Japan") OR (MH "South Korea") OR (MH "Austria") OR (MH "Belgium") OR (MH "Estonia") OR (MH "Latvia") OR (MH "Lithuania") OR (MH "Czech Republic") OR (MH "Hungary") OR (MH "Poland") OR (MH "Slovakia") OR (MH "Slovenia") OR (MH "France") OR (MH "Germany") OR (MH "United Kingdom") OR (MH "England") OR (MH "Greece") OR (MH "Ireland") OR (MH "Italy") OR (MH "Luxembourg") OR (MH "Netherlands") OR (MH "Portugal") OR (MH "Denmark") OR (MH "Iceland") OR (MH "Norway") OR (MH "Sweden") OR (MH "Spain") OR (MH "Switzerland") OR (MH "Australia") OR (MH "New Zealand")
9. TI canad* OR AB canad*
10. TI british columbia OR AB british columbia
11. TI alberta OR AB alberta
12. TI saskatchewan OR AB saskatchewan
13. TI manitoba OR AB manitoba
14. TI ontario OR AB ontario
15. TI quebec OR AB quebec
16. TI new brunswick OR AB new brunswick
17. TI nova scotia OR AB nova scotia
18. TI prince edward island OR AB prince edward island
19. TI (newfoundland and labrador) OR AB (newfoundland and labrador)
20. TI nunavut OR AB nunavut
21. TI northwest territories OR AB northwest territories
22. TI yukon OR AB yukon
23. TI high N2 income N2 countr* or AB high N2 income N2 countr*
24. (MH "Developed Countries")
25. TI reception N2 countr* or AB reception N2 countr*
26. TI resettlement N2 countr* or AB resettlement N2 countr*
27. (MH "Health")
28. TI health N2 need* or AB health N2 need*
29. TI health N2 care N2 need* or AB health N2 care N2 need*
30. TI unmet N2 need* or AB unmet N2 need*
31. (MH "Health Services Needs and Demand")
32. (MH "Health Care Delivery")
33. (MH "Transcultural Care")
34. (MH "Health Services Accessibility")
35. (MH "Health Status")
36. (MH "Health Status Disparities")
37. (MH "Healthcare Disparities")
38. (MH "Needs Assessment")
39. (MH "Occupational Health")
40. TI barrier* or AB barrier*
41. TI challenge* or AB challenge*
42. TI constraint* or AB constraint*
43. S1 OR S2 OR S3 OR S4 OR S5 OR S6 OR S7
44. S8 OR S9 OR S10 OR S11 OR S12 OR S13 OR S14 OR S15 OR S16 OR S17 OR S18 OR S19 OR S20 OR S21 OR S22 OR S23 OR S24 OR S25 OR S26
45. S27 OR S28 OR S29 OR S30 OR S31 OR S32 OR S33 OR S34 OR S35 OR S36 OR S37 OR S38 OR S39 OR S40 OR S41 OR S42
46. S43 AND S44 AND S45
47. S43 AND S44 AND S45 Limiters - Published Date: 20110101-20210731; English Language

Embase Search String

1. 'refugee'/exp
2. refugee*:ab,kw,ti
3. (asylum NEAR/2 seeker*):ab,kw,ti
4. 'forced migrant'/de
5. (temporary NEAR/2 foreign NEAR/2 worker*):ab,ti,kw
6. 'foreign worker'/de
7. 'migrant worker'/exp
8. (foreign NEAR/2 worker*):ab,ti,kw
9. (migrant NEAR/2 worker*):ab,ti,kw
10. 'canada'/exp OR 'united states'/exp OR 'chile'/exp OR 'israel'/de OR 'japan'/exp OR 'south korea'/exp OR 'austria'/exp OR 'belgium'/de OR 'estonia'/exp OR 'latvia'/exp OR 'lithuania'/exp OR 'czech republic'/exp OR 'hungary'/exp OR 'poland'/exp OR 'slovakia'/exp OR 'slovenia'/exp OR 'france'/de OR 'germany'/de OR 'united kingdom'/de OR 'england'/exp OR 'greece'/exp OR 'ireland'/exp OR 'italy'/exp OR 'luxembourg'/exp OR 'netherlands'/exp OR 'portugal'/de OR 'denmark'/exp OR 'finland'/de OR 'iceland'/exp OR 'norway'/de OR 'sweden'/exp OR 'spain'/de OR 'switzerland'/exp OR 'australia'/de OR 'new zealand'/exp
11. canad*:ab,kw,ti
12. 'british columbia':ab,kw,ti
13. alberta:ab,kw,ti
14. saskatchewan:ab,kw,ti
15. manitoba:ab,kw,ti
16. ontario:ab,kw,ti
17. quebec:ab,kw,ti
18. 'new brunswick':ab,kw,ti
19. 'nova scotia':ab,kw,ti
20. 'prince edward island':ab,kw,ti
21. 'newfoundland and labrador':ab,kw,ti
22. 'nunavut':ab,kw,ti
23. 'northwest territories':ab,kw,ti
24. 'yukon':ab,kw,ti
25. 'high income country'/exp
26. 'developed country'/exp
27. (reception NEAR/2 countr*):ab,kw,ti
28. (resettlement NEAR/2 countr*):ab,kw,ti
29. 'health'/de
30. 'unmet needs'/exp
31. 'health care need'/de
32. 'health care delivery'/de
33. 'transcultural care'/de
34. 'health care access'/de
35. 'health status'/de
36. 'health disparity'/exp
37. 'health care disparity'/de
38. 'needs assessment'/de
39. 'occupational health'/de
40. 'unmet medical need'/exp
41. 'medical care need'/exp
42. 'barrier'/exp
43. 'challenge'/exp

44. 'constraint'/exp
45. (health NEAR/2 need*):ab,kw,ti
46. (unmet NEAR/2 health NEAR/2 need*):ab,kw,ti
47. barrier*:ab,kw,ti
48. challenge*:ab,kw,ti
49. constraint*:ab,kw,ti
50. #1 OR #2 OR #3 OR #4 OR #5 OR #6 OR #7 OR #8 OR #9 OR
51. #10 OR #11 OR #12 OR #13 OR #14 OR #15 OR #16 OR #17 OR #18 OR #19 OR #20 OR #21 OR #22 OR #23 OR #24 OR #25 OR #26 OR #27 OR #28
52. #29 OR #30 OR #31 OR #32 OR #33 OR #34 OR #35 OR #36 OR #37 OR #38 OR #39 OR #40 OR #41 OR #42 OR #43 OR #44 OR #45 OR #46 OR #47 OR #48 OR #49
53. #50 AND #51 AND #52
54. #50 AND #51 AND #52 AND [english]/lim AND [2011-2021]/py
